# MicroRNAs in metabolism

**DOI:** 10.1111/apha.12681

**Published:** 2016-04-05

**Authors:** S. Vienberg, J. Geiger, S. Madsen, L. T. Dalgaard

**Affiliations:** ^1^ Center for Basic Metabolic ResearchFaculty of HealthUniversity of CopenhagenCopenhagenDenmark; ^2^ Department of Science and EnvironmentRoskilde UniversityRoskildeDenmark

**Keywords:** adipocytes, metabolism, microRNA, non‐alcoholic hepato‐steatosis, type 2 diabetes mellitus, *β*‐cells

## Abstract

MicroRNAs (miRNAs) have within the past decade emerged as key regulators of metabolic homoeostasis. Major tissues in intermediary metabolism important during development of the metabolic syndrome, such as *β*‐cells, liver, skeletal and heart muscle as well as adipose tissue, have all been shown to be affected by miRNAs. In the pancreatic *β*‐cell, a number of miRNAs are important in maintaining the balance between differentiation and proliferation (miR‐200 and miR‐29 families) and insulin exocytosis in the differentiated state is controlled by miR‐7, miR‐375 and miR‐335. MiR‐33a and MiR‐33b play crucial roles in cholesterol and lipid metabolism, whereas miR‐103 and miR‐107 regulates hepatic insulin sensitivity. In muscle tissue, a defined number of miRNAs (miR‐1, miR‐133, miR‐206) control myofibre type switch and induce myogenic differentiation programmes. Similarly, in adipose tissue, a defined number of miRNAs control white to brown adipocyte conversion or differentiation (miR‐365, miR‐133, miR‐455). The discovery of circulating miRNAs in exosomes emphasizes their importance as both endocrine signalling molecules and potentially disease markers. Their dysregulation in metabolic diseases, such as obesity, type 2 diabetes and atherosclerosis stresses their potential as therapeutic targets. This review emphasizes current ideas and controversies within miRNA research in metabolism.

For the past 50 years, the term ‘gene’ has been synonymous with regions of the genome encoding mRNAs that are translated into protein. However, the past decade's explosion of large‐scale genome sequencing has revealed that opposed to the original expectation that more complex organisms would have a greater number of genes, it is now clear that human and mice shares a similar number of protein encoding genes as the round worm *C. elegans*. A possible explanation for this paradox comes from the insight that biological complexity generally correlates with proportion of the genome which is non‐protein‐coding (Taft *et al*. 2007). The majority of this non‐protein‐coding region is transcribed into long non‐coding RNA or small non‐coding RNA, which orchestrate the regulation of protein expression both at the transcriptional and translational level. A class of small non‐coding RNAs termed microRNAs (miRNAs) was discovered in 1993 by Lee, Feinbaum and Ambros (Lee *et al*. 1993). MiRNAs consist of approx. 22 nucleotides and regulate gene expression by binding to their complementary sites within the 3′‐untranslated regions (3′UTRs) of target mRNAs (Lagos‐Quintana *et al*. 2001) resulting in mRNA translational repression or transcript degradation. The degree of miRNA‐target base‐pairing complementarity determines the fate of the target transcript. Perfect complementarity leads to target cleavage and degradation. In contrast, imperfect complementarity triggers mRNA silencing by distinct mechanisms which may involve translational repression, slicer‐independent mRNA degradation and/or sequestration in cytoplasmic processing bodies (Roberts 2015) (Fig. 1).

**Figure 1:**
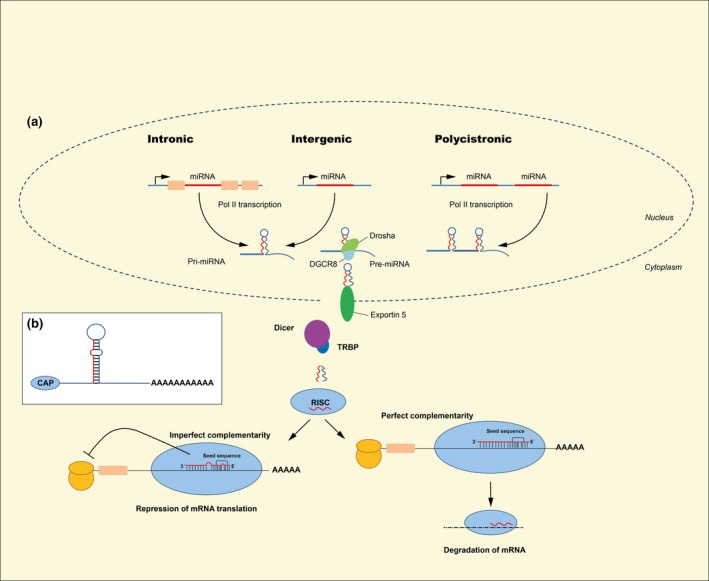
The canonical miRNA biogenesis pathway (a) and the average precursor miRNA (b). (a): The miRNA genes lies either intronic, intergenic or polycistronic. The primary miRNA (Pri‐miRNA) is transcribed by polymerase II (or polymerase III). The Pri‐miRNA is cleaved by the microprocessor complex Drosha‐DGCR8 in the nucleus. The precursor miRNA (pre‐miRNA) is transported out in the cytoplasm by Exportin 5. In the cytoplasm, the pre‐miRNA is further cleaved to its mature length (approx. 22 nt) by the RNase III Dicer in complex with the double‐stranded RNA‐binding protein TRBP. Argonaute (AGO2) proteins unwind the miRNA duplex and facilitate incorporation of the guide strand (red) into the RNA‐induced silencing complex (RISC). AGO2 then guides the RISC miRNA assembly to target mRNAs, whereas the passenger strand (blue) is degraded. Some miRNA bind mRNA with perfect complementarity and induce degradation of mRNA. miRNA also bind to targets with imperfect complementarity and block translation. (b): An average precursor miRNA with a hairpin stem of 33 base pairs, a terminal loop and two flanking region, where 5′ end are capped and a polyadenylated 3′ end.

Each miRNA may have hundreds of mRNA targets, just as well as a single mRNA may be regulated by several distinct miRNAs adding to the layer of complexity of protein expression. MiRNAs are encoded in diverse regions of the genome including non‐coding regions (intronic or intergenic) as well as protein coding (in exons). The canonical biogenesis of the mature functional miRNAs involves multiple processing steps, described in Figure 1. Each processing step contains another layer of regulation and therefore adds to the complexity of gene expression.

The aim of this review is to highlight the recent progress and challenges within research of miRNAs in metabolism and metabolic disease, with a special emphasis on specific and major tissues and cell types important for development of the metabolic syndrome, obesity and type 2 diabetes: *β*‐cells, liver, skeletal and heart muscle and adipose tissue. Although organ to organ crosstalk greatly impacts metabolism, it is not the scope of this review. Furthermore, we also describe recent progress on development of miRNA therapeutics and biomarkers, as well as challenges in quantification of miRNAs.

## Pancreatic islets and β‐cells

The islet of Langerhans constitutes an important node of control for maintaining normoglycaemia, as sufficient *β*‐cell insulin secretion is needed for proper peripheral glucose uptake, and *α*‐cell glucagon secretion is important for hepatic glucose production. The importance of *β*‐cells for glucose homoeostasis is underlined by the observation that type 2 diabetes mellitus only develops in the context of *β*‐cell failure. Moreover, genetic studies reveal that amongst the more than 75 genetic loci associating with type 2 diabetes, the largest proportion harbours transcripts important for *β*‐cell function or proliferation (Rutter 2014).

The level of gene expression or function is regulated at many levels, and miRNA networks constitute control points for integration of environmental and genetic factors influencing physiological responses of the *β*‐cell. In general, most genetics studies with miRNA mutants display no obvious phenotype unless the animal is confronted by physiological stressors. For *β*‐cells, increased stress occurs for example as their workload increases due to peripheral insulin resistance, which may result in progression to type 2 diabetes (Halban *et al*. 2014).

Evidence to support an important role of miRNAs in *β*‐cells has been obtained from cre‐mediated deletion of *Dicer1* in different pancreatic lineages. Pdx1‐cre‐mediated *Dicer1* deletion shows that developmentally expressed miRNAs are important for proper islet and *β*‐cell development (Lynn *et al*. 2007) and both induced and constitutive *Dicer1* deletion in *β*‐cells results in impaired insulin secretion and diabetes (Kalis *et al*. 2011, Melkman‐Zehavi *et al*. 2011, Martinez‐Sanchez *et al*. 2015) with impaired glucose‐stimulated insulin secretion (GSIS) preceding changes in insulin content or *β*‐cell mass.

Another important role of miRNAs is thought to be through selective repression of mRNAs, whose expression is detrimental to correct functioning of specific cell types, termed ‘disallowed’ genes. For *β*‐cells *Slc16a1* (pyruvate transporter), *Ldha* (lactate dehydrogenase A)*, Fcgrt1* (Neonatal Fc receptor), *Pdfgra* (PDGF receptor, alpha‐type) and *Oat* (ornithine aminotransferase) are selectively repressed by miRNAs, as these mRNAs are de‐repressed in *dicer*1 KO islets and their 3′UTRs confer increased reporter‐gene activity in *dicer1* depleted islet cells (Pullen *et al*. 2011, Martinez‐Sanchez *et al*. 2015). The Argonaute 2 (Ago2) component of the RISC complex controls compensatory *β*‐cell proliferation and is under the control of miR‐184, which is negatively regulated by blood glucose levels, thus providing a systemic feedback to the *β*‐cells reflecting systemic insulin resistance (Tattikota *et al*. 2014, 2015).

Although very few miRNAs are tissue specific, a number of miRNAs can be designated as being either enriched in endocrine, neuro‐endocrine or epithelial tissues, where the miR‐375 belongs to the endocrine enriched miRNAs (10% of *β*‐cell microRNA is miR‐375) (Poy *et al*. 2004, van de Bunt *et al*. 2013), the miR‐7 family to the neuro‐endocrine, and the 200‐family is expressed in epithelial tissues from which islet cells originate. The phenotype of the global miR‐375 knockout (KO) (Poy *et al*. 2009) shows progressive hyperglycaemia with lower numbers of *β*‐cells and impaired compensatory *β*‐cell proliferation as well as effects on Na^+^ channel inactivation properties (Salunkhe *et al*. 2015a). Thus, the miR‐375 deficient *β*‐cells' adaptation to stress and insulin resistance resembles a phenocopy of *Dicer1*‐KO *β*‐cells (Lynn *et al*. 2007). Conversely, specific *β*‐cell re‐expression of miR‐375 in the KO mouse normalizes glucose tolerance. Although miR‐375 is the most highly expressed miRNA in pancreatic *β*‐cells, under normal physiological conditions, only 1% of plasma miR‐375 is derived from *β*‐cells, which only doubles after streptozotocin induced diabetes (Latreille *et al*. 2015). Thus, these findings challenge the use of miR‐375 as a circulating biomarker for *β*‐cell injury (Erener *et al*. 2013).

Whereas miR‐375 expression is necessary for correct *β*‐cell function and proliferation, targeted deletion of either miR‐7 or miR‐200 family members display improved *β*‐cell function in mice fed a normal diet indicating that the role of these miRNAs are to constitutively repress *β*‐cell function (Latreille *et al*. 2014, Belgardt *et al*. 2015). Conditional *β*‐cell KO of all three miRNAs of the miR‐7 family preferentially changed the abundance of synaptic proteins involved in exocytosis, for which target gene de‐repression was observed and insulin exocytosis was increased (Latreille *et al*. 2014). Whether the miR‐7 family is regulated the same way in other tissues with high rate of exocytosis still needs to be examined. The miR‐200 family consists of miR‐200a/141 and miR‐200b/miR‐200c/miR‐429 clusters. These miRNAs are generally dysregulated in cancers, but miRNAs belonging to these subfamilies are also very abundant in *β*‐cells and amount up to 2/3 of all miRNAs in *β*‐cells. They are decreased by high fat diet and upregulated in diabetic db/db (BKS background) about threefold. Furthermore, forced expression of the miR‐141/200a cluster by just fivefold in *β*‐cells results in overt diabetes and subsequent death of the mice and is accompanied by massive *β*‐cell apoptosis (Belgardt *et al*. 2015). The double KO of miR‐141/200a and 200b/c/429 clusters protects against *β*‐cell ER‐stress in the Akita mouse model, which as misfolding of insulin, as well as in both multiple low‐dose streptozotocin and single‐dose streptozotocin induced *β*‐cell damage. The *β*‐cell protection is mediated through regulation of *Tp53* activity. Thus, two large families of miRNAs negatively control *β*‐cell function and survival. One could ask why the pancreatic *β*‐cells are under such heavy negative control. In the setting of limited access to nutrients, having excessive insulin production and secretion would be detrimental to an individual due to the risk of hypoglycaemia. Consequently, there will be a high selective pressure to develop mechanisms keeping *β*‐cells under control.

However, the environment to which most subjects are exposed is not one of famine but rather one of feast. Therefore, it is important to study *β*‐cell failure in type 2 diabetes mellitus and the impact of environment on islet response and impaired insulin secretion. Insulin exocytosis is a very specialized feature of *β*‐cells. It is affected by several diabetes associated gene variants, but the expression of exocytotic genes has been shown to be reduced islets from patients with type 2 diabetes, which is not due to genetic variation in the vicinity of these genes. Epigenetic variation, such as DNA methylation (Dayeh *et al*. 2014) or miRNAs, may mediate some of the expression changes. In an attempt to identify miRNAs involved in *β*‐cell decompensation, islets were isolated from Goto Kakizaki (GK)‐rats, a model of type 2 diabetes with *β*‐cell dysfunction. miR‐335, miR‐152 and miR‐130a/b were found to be increased (Esguerra *et al*. 2011) and computational analysis showed that miR‐335 targeted transcripts encoding exocytotic proteins (*Stxbp1*,* Syt11*,* Snap25*). Moreover, overexpression of miR‐335 leads to decreased GSIS as well as decreased depolarization‐induced insulin exocytosis (Salunkhe *et al*. 2015b). Additionally, the poor insulin‐secretor cell line INS‐832/2 had increased levels of miR‐152 and miR‐130a/b compared with INS‐832/13 cells, which maintain a high GSIS (Hohmeier *et al*. 2000, Ofori *et al*. 2014). In line with this, knockdown of miR‐152 leads to increased GSIS, whereas overexpression impaired GSIS in the INS‐832/13 cells, with a concomitant decrease in insulin content (Ofori *et al*. 2014). Moreover, miR‐187 was shown to be increased in human type 2 diabetic islets, negatively correlating with GSIS in islets from normoglycaemic donors, and forced expression in rat islets and INS‐1 cells reduced GSIS (Locke *et al*. 2014). Thus, multiple miRNAs participate in and control insulin exocytosis, are dysregulated in type 2 diabetic islets and constitute natural nodes in cellular interaction networks regulating insulin exocytosis (Eliasson 2014).

*β*‐cells have low proliferation rates, which decrease even further as an animal age (Wang *et al*. 2015b). However, mature *β*‐cells release more insulin in response to glucose than *β*‐cells from young animals (Jacovetti *et al*. 2015), making it attractive to understand how a *β*‐cell can be kept ‘young’ in terms of proliferative capacity and ‘old/mature’ in terms of insulin secretion. When rat pup islets from 10 days of age (D10) are compared with adult islets, proliferation rate is higher and the level of GSIS is lower, while insulin content is unchanged. The transition was found to occur 2–5 days following weaning, and the associated change in nutrients is thought to induce the shift between proliferation rate and GSIS. Rats prematurely weaned showed the same metabolic shift and maturation, further indicating that it is a change in nutrients that drive the shift. Interestingly, several miRNAs were altered in the young vs. the mature islets. For example, were the miR‐29 family, miR‐204 and miR‐129 upregulated, whereas the miR‐17‐92 cluster, miR‐181b and miR‐215 were suppressed more than twofold from young (D10) to adult islets. The same expression pattern was present in prematurely weaned pups. Surprisingly, when pups were fed a high fat diet the transition into high GSIS did not occur and this was associated with an extended immature miRNA profile. To investigate the contribution of single miRNAs to this phenotype, D10 isolated islets were dissociated into single islet cells, transfected using antisense oligonucleotides targeting miRNAs or miRNA mimetics and GSIS and *β*‐cell proliferation were determined. Using this approach, both the miR‐17‐92 cluster and miR‐181b were found to regulate *β*‐cell replication as well as GSIS. Using luciferase reporter assays, the miR‐17‐92 cluster was shown to target 3′UTRs of *Pfkp* (phosphofructokinase, platelet), *Tgfbr2* (transforming growth factor beta receptor II) and *Pten* (phosphatase and tensin homolog), while miR‐181b targeted *Gpd2* (glycerol‐3‐phosphate dehydrogenase 2), *Mdh1* (malate dehydrogenase 1) and *Sirt1* (sirtuin 1). Of note, the miR‐17‐92 cluster is also a known transcriptional target of E2F and regulates cMyc levels in other cell types (Aguda *et al*. 2008). It seems likely that a similar pathway could control the decrease in *β*‐cell proliferation occurring with weaning and maturation. *β*‐cell proliferation is decreased by ageing, which is partly due to decreased amounts of *Pdgfra*, mediated by an ageing‐induced increase in miR‐34a (Tugay *et al*. 2015). From these studies, it is clear that nutritional state, malnutrition and foetal programming affect the maturity state of the *β*‐cells. However, how this translates into human subjects and human nutrition, and for example, the role of infant formula compared to breast milk feeding is not known and will need to be addressed.

## Liver metabolism regulated by miRNAs

The liver plays a major role in energy metabolism as it is a main contributor to absorptive glucose storage and post‐absorptive glucose release, amino acid metabolism and is the main regulator of lipoprotein metabolism. Although functions of miRNAs were first described for the regulation of proper development of *C. elegans* (Lee *et al*. 1993), several studies have revealed that miRNAs play a pivotal role for controlling metabolic homoeostasis. miR‐122 is the most abundant miRNA in liver and has been shown to be involved in hepatic cholesterol and lipid metabolism (Krützfeldt *et al*. 2005). Two studies have shown that antisense targeting miR‐122 results in significant reduction in plasma cholesterol levels (Krützfeldt *et al*. 2005, Esau *et al*. 2006), but the effect on cholesterol metabolism by miR‐122 seems to be indirect, as the exact targets of this particular miRNA are still unclear (Fernandez‐Hernando *et al*. 2013). Another miRNA implicated in hepatic cholesterol metabolism is miR‐33 originating from two intronic miRNAs, miR‐33a and miR‐33b, which are encoded within the introns of *Srebf2* and *Srebf1* genes respectively (Najafi‐Shoushtari *et al*. 2010). Both miRNAs are co‐transcribed with their host genes and under their regulation. MiR‐33a directly targets the cholesterol transporters *Abca1* and *Abcg1*, which are responsible for the efflux of cholesterol from the cell, suggesting the importance of this miRNA in cholesterol metabolism. In agreement with this, the miR‐33a KO mouse has shown an increase in *Abca1* expression and plasma HDL levels (Horie *et al*. 2010). The data are supported by three independent studies using different strategies to inhibit endogenous miR‐33a, which also increase plasma HDL levels (Marquart *et al*. 2010, Najafi‐Shoushtari *et al*. 2010, Rayner *et al*. 2010). Besides cholesterol metabolism miR‐33b has also been shown to be implicated in fatty acid *β*‐oxidation, as carnitine palmitoyltransferase (*Cpt1a*) is regulated by miR‐33b. Interestingly, miR‐33 is also highly abundant in brain (Rayner *et al*. 2010), and it has previously been shown that the cholesterol metabolism in brain of diabetic animals is indeed compromised (Suzuki *et al*. 2010), suggesting a potential role for miR‐33 in brain cholesterol metabolism.

In an effort to systematically identify miRNAs that regulate cholesterol metabolism through the low‐density lipoprotein receptor (LDLR), Goedeke *et al*. (2015) developed a high‐throughput screen to monitor the effect of miRNAs to induce cellular low‐density lipoprotein (LDL) uptake. During this screen, miR‐148a was discovered as a top candidate for LDLR regulation. And indeed, when miR‐148a is suppressed, LDL levels decrease, whereas HDL levels increase, highlighting the therapeutic potential for the treatment of atherosclerosis and related dyslipidaemias of this particular miRNA.

Several other miRNAs have been shown to be implicated in liver metabolism and glucose homoeostasis. miR‐143 (Jordan *et al*. 2011), miR‐181a (Zhou *et al*. 2012), miR‐103 and miR‐107 (Trajkovski *et al*. 2011) have all been shown to affect hepatic insulin sensitivity, and more recently, miR‐802 has been shown to be increased with obesity and that its reduction improves glucose tolerance and insulin action (Kornfeld *et al*. 2013). From the above studies, it is clear that miRNAs play a central role in regulation of liver metabolism and most likely more metabolically important miRNAs will be discovered in the future and will serve as potential targets for treatment of metabolic disorders. However, using miRNAs as therapeutics faces numerous challenges, which will be discussed in the section of ‘*miRNAs as therapeutics’*.

## Muscle and heart miRNAs

Skeletal muscle accounts for more than 40% of the body weight of a normal healthy person and is by far the largest organ of the body. Furthermore, impaired insulin‐stimulated muscle glucose disposal is the primary defect in the insulin resistant state during a hyperinsulinaemic–euglycaemic clamp (DeFronzo *et al*. 1985), highlighting the importance of skeletal muscle in glucose homoeostasis. Besides their importance in whole‐body glucose homoeostasis, skeletal muscles also play a pivotal role in healthy ageing, and muscle wasting is present in many diseases, such as sarcopenia, HIV–AIDS, cancer cachexia, renal failure and bed rest (Bodine 2013, Polge *et al*. 2013), showing the importance of development and maintenance of muscle mass. MiRNAs have been shown to be necessary for appropriate muscle development, as muscle specific dicer1 knockout mice have marked dysregulation of muscle development resulting in embryonic lethality (O'Rourke *et al*. 2007). In addition, miRNAs are involved in control of muscle fibre type (Van Rooij *et al*. 2009), namely miR‐208a, miR‐208b and miR‐499, with each of these miRNAs encoded in the myosin heavy chain (MHC) genes (McCarthy *et al*. 2009) signifying their importance in muscle phenotype. In agreement, miR‐208b and miR‐499 are decreased concomitant with the expression of the respective MHC genes, inversely correlated with myostatin in human skeletal muscle after spinal cord injury, and thereby linked to the regulation of muscle mass (Boon *et al*. 2015). In line with this, age‐related changes in miR‐143 have recently been shown to affect muscle regeneration *in vitro* (Soriano‐Arroquia *et al*. 2016).

Several miRNAs are enriched in muscle and heart (Sempere *et al*. 2004), and especially, miR‐1, miR‐133 and miR‐206 are defined as myogenic miRNAs capable of inducing skeletal muscle differentiation in murine models (Chen *et al*. 2006, Dey *et al*. 2011). To address the miRNA network of human skeletal muscle cell differentiation, Sjögren and colleagues performed a time course analysis from day 0 (myoblast) to day 10 (myotube) coupled to microarray analyses to identify differential miRNA and mRNA expression (Sjogren *et al*. 2015). Confirming data from murine muscle cells largest fold change in miRNA expression were indeed observed for miR‐1, miR‐133a, miR‐133b and miR‐206. Integrating the miRNAs and mRNAs that were differentially expressed in a network analysis pointed to nodes of miRNA regulation containing genes such as *HEYL*,* NR4A2*,* NR4A3*,* PAX7* and *PHIP*. Interestingly, these are all annotated as regulators of muscle developments and differentiation. Moreover, functional studies with overexpression of miR‐30b, also differentially expressed during muscle differentiation, showed that not all *in‐silico* predicted gene targets were necessarily regulated by manipulation of this particular miRNA. Despite the integration of simultaneous miRNA and mRNA, expression data with target prediction algorithms and network this study clearly show that bioinformatics‐based deductions cannot substitute for experimental validation of miRNA function (Sjogren *et al*. 2015). Therefore, hypothesis‐driven functional studies of miRNA and target genes within skeletal muscle development, as well as other biological settings, still needs to be addressed.

The muscular walls of the heart (the myocardium) are responsible for pumping blood through the lungs and the rest of the body, and it is clearly important that the muscles in the heart maintain their activity under all conditions. The increased risk of premature death with type 2 diabetes is not because of diabetes per se, but the cardiovascular diseases and their comorbidities which follow with the development of diabetes and obesity. Microvascular complications occur as long‐term sequelae to poorly controlled type 2 diabetes and include diabetic retinopathy, neuropathy and impaired wound healing as well as nephropathy. The role of miRNAs and their impact on microvascular complications has been excellently covered elsewhere (Moura *et al*. 2014, Banerjee & Sen 2015, Bhatt *et al*. 2016).

Several miRNAs are enriched in the heart (Sempere *et al*. 2004), and heart‐specific dicer1 KO mice die young by severe heart failure mainly characterized by hypertrophic growth (Chen *et al*. 2008) demonstrating the importance for Dicer in cardiac contraction and indicate that miRNAs play a key role in proper heart function. In humans, cardiac hypertrophy is the main risk factor for the development of heart failure and lethal arrhythmias (Towbin & Bowles 2002) and is usually linked to hypertension and macrovascular complications. The development of cardiac hypertrophy is linked to aberrant reactivation of embryonic gene programmes, which is still not completely understood. It is known, however, that transcription factors sharing a basic helix‐loop‐helix domain are important for determination and differentiation of various cell types, including cardiomyocytes. Elegantly, Dirkx and colleagues (Dirkx *et al*. 2013) showed that such a transcription factor, heart and neural crest derivatives expressed transcript (*Hand2*) which is required for proper heart development (Hutson & Kirby 2007, Snider *et al*. 2007), is re‐activated in the failing heart, where it drives the induction of a gene network controlling cardiac growth, dilation and dysfunction. Interestingly, *Hand2* is inversely expressed with miR‐1, miR‐92a, miR‐92b and miR‐25 in experimental models of heart disease and *in vivo* inhibition of miR‐25 resulted in cardiac dysfunction in a *Hand2* dependent manner (Dirkx *et al*. 2013). Several other miRNAs have been shown to be implicated in pathological cardiac hypertrophy including miR‐133 (Care *et al*. 2007), miR‐199b (Da Costa Martins *et al*. 2010) and miR‐378 (Ganesan *et al*. 2013) signifying the potential for targeting specific miRNAs with RNA based therapeutics.

## MiRNAs in adipose tissue and adipocyte differentiation

The traditional view of adipose tissues as biologically inactive lipid storage depots has changed over the course of the last 30 years. It is now clear that the adipose tissues are highly responsive endocrine organs that influence metabolic homoeostasis and inflammation (Rosen & Spiegelman 2006). The importance of adipose tissue function in health and disease is revealed by the range of diseases previously associated with ageing that are more prevalent amongst overweight and obese individuals. The discovery of an age‐induced decline in miRNA processing, specifically in the adipose tissue, underlines the importance of this tissue. The abundance of the key miRNA processing enzyme, Dicer, is reduced in white adipose tissue (inguinal and perigonadal) by ageing and followed by a coordinated decline in levels of multiple miRNAs, an observation conserved between mice, human and nematodes. In mice subjected to calorie restriction, Dicer levels do not decline (Mori *et al*. 2012b). Knockdown of Dicer in cells results in premature senescence, and mice deficient of Dicer in adipose tissue develop lipodystrophic loss of intra‐abdominal and subcutaneous white fat, severe insulin resistance and enlargement and ‘whitening’ of intrascapular brown fat (Mori *et al*. 2012b, 2014), and miR‐365 was identified as a miRNA that partially can explain the ‘whitening’ phenotype.

Brown adipose tissue (BAT) has an obvious therapeutic potential, and many studies highlight the importance of miRNAs in the formation of BAT, such as miR‐193b/‐365 (Sun *et al*. 2011, Feuermann *et al*. 2013), miR‐196a (Mori *et al*. 2012a), miR‐155 (Chen *et al*. 2013) and miR‐133a/b (Trajkovski *et al*. 2012). Recently, Zhang *et al*. showed by combining miRNA and mRNA microarray data that miR‐455 plays a crucial role in brown adipogenesis. MiR‐455, a BMP7‐induced miRNA, targets several key adipogenic regulators, such as Necdin and Runx1t1, which are important adipogenic suppressors gating an adipocyte differentiation programme. In addition, miR‐455 targets hypoxia‐inducible factor 1a inhibitor (HIF1an), a hydroxylase that normally inhibits AMPK activity by hydroxylation, which leads to AMPK activation. Thus, miR‐455 suppresses Necdin and Runx1t1 to initiate adipogenic programme and suppresses HIF1an to activate AMPK which, in turn, acts as a metabolic trigger to induce a brown adipogenesis (Zhang *et al*. 2015). It is indeed interesting to observe that BAT expressed miRNAs to a certain degree reflect the myogenic lineage of these cells in that BAT shares important functional miRNAs with skeletal muscle (Table 1).

**Table 1:** MiRNAs of importance for metabolically relevant tissues

*β*‐cells	Liver	Sk. Muscle	Heart	WAT	BAT
miR‐375	miR‐122	miR‐1	miR‐25	miR‐365	miR‐365
miR‐200 family	miR‐33a/b	miR‐206	miR‐199a‐214	miR‐193b	miR‐193b
miR‐7 family	miR‐148a	miR‐208b	miR‐155	miR‐126	miR‐155
miR‐335	miR‐143	miR‐133a/b		miR‐92a	miR‐133a/b
miR‐152	miR‐103/107	miR‐499			miR‐196a
miR‐29 family	miR‐802	miR‐30b			miR‐455
miR‐181b	miR‐181a	miR‐143			
miR‐184					
miR‐187					
miR‐204					
miR‐17‐92					
miR‐129					
miR‐34a					
miR‐215					
miR‐130a/b					

MiRNAs in this table are all cited in the text and is not an exhaustive list of all miRNAs shown to be important for the function of these tissues. MiRNAs are given by number only, for identity of 5′ or 3′ mature species please refer to the original publication.

Several studies have compared miRNA expression profiles in obese and lean white adipose tissue from mice and from humans. In fat cells from mice with diet‐induced obesity, 35 of the 574 detected miRNAs were differentially expressed (Xie *et al*. 2009). Microarray screening of human WAT tissues identified a number of miRNAs that are potentially dysregulated in patients with obesity, both in those with concurrent type 2 diabetes mellitus and in glucose tolerant subjects (Heneghan *et al*. 2011, Keller *et al*. 2011). However, it has proven difficult to validate reported miRNAs in other cohorts, as well as the direction of expression of the miRNA between lean and obese groups, all of which is excellently reviewed in Arner & Kulyte (2015) (Arner & Kulyte 2015). This highlights the challenge that many miRNA studies are difficult to reproduce. As possible sources of variation, one has to consider the sample origin, which tissue or cell type is examined, the gender of test subjects and disease state. Furthermore, many microarray studies are often underpowered, which also contributes to ambiguous results (Witwer 2013), not to mention which platform was used to detect the miRNAs (Mestdagh *et al*. 2014).

MiRNAs often assert their action in families and thus work in networks. Arner *et al*. (2012) found that 11 miRNAs were downregulated in obese subjects, all of which target the expression of CCL2 (C‐C motif ligand 2, also known as MCP1 (macrophage chemoattractant protein 1) (Arner *et al*. 2012). The chemokine CCL2 is suspected to initiate inflammation in the adipose tissue, which in turn is could be a driver of whole‐body insulin resistance. Also, it is known that obese individuals have an increased secretion of CCL2 compared to lean controls (Arner *et al*. 2012). A subnetwork involving miR‐126, miR‐193b and miR‐92a was shown to associate inversely with transcription factors controlling inflammation in the adipose tissue and that the regulation of these miRNAs had an additive effect on CCL2 secretion (Kulyte *et al*. 2014). Even though there is conflicting data, especially in the context of WAT and miRNAs, accumulating data indicate that miRNAs are central modulators of normal WAT and BAT differentiation and biology (Rottiers & Naar 2012).

## MiRNAs as therapeutics

**Figure 2:**
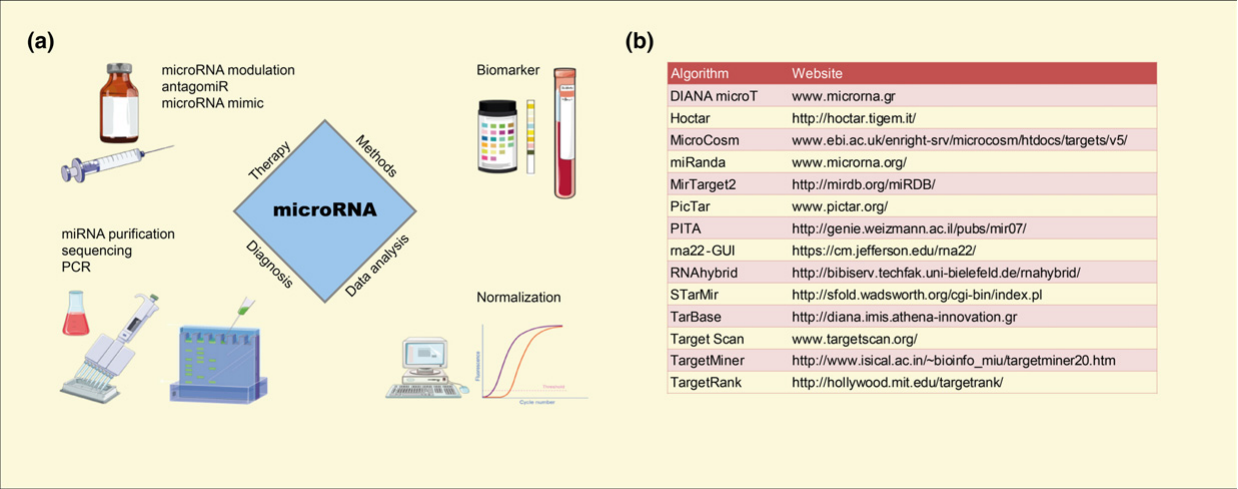
(a) Translational aspects of miRNAs. For therapy, miRNAs may be used as antagonists (antagomiR) or agonists (microRNA mimics). Specific methods, such as next‐generation sequencing of small RNAs and qPCR‐based miRNA arrays are used to identify possible miRNA biomarkers from patient specimens, but may also be used to monitor therapeutic effects or unintended side effects by treatment. Integral to the translational use of miRNAs are improved and consistent data analysis strategies of qPCR results with special focus on using optimal data normalization and identification of proper reference genes or miRNAs. For these purposes, current techniques and analysis strategies need to be adjusted and optimized. The figure was assembled with the help of Servier Medical Art (http://www.servier.com/Powerpoint-image-bank). (b) Table of Web‐tools commonly used for prediction of miRNA‐target mRNAs.

The growing knowledge about miRNAs and their molecular actions gives rise to innovative industrial applications for this class of molecules (Fig. 2a). Amongst the most promising perspectives are the usage of miRNAs in medical therapy and their potential as new biomarkers (Hayes *et al*. 2014). Both topics will be discussed in the following section with focus on current challenges, existing solution strategies and future perspectives.

Given that miRNAs are involved in regulation of a magnitude of cellular processes (Esau *et al*. 2006, Trajkovski *et al*. 2011, Rottiers & Naar 2012, Dumortier *et al*. 2013), it is not surprising that miRNA expression patterns change in obesity (Ortega *et al*. 2013) and diabetes (Kong *et al*. 2010, Hayes *et al*. 2014, Yang *et al*. 2014). Pathophysiologically, upregulated miRNAs can be manipulated by complementary nucleotide analogues to decrease their effective activity (Stenvang *et al*. 2012). These ‘antagomiRs’ often have a modified nucleotide architecture, where, for example, the nucleic acid ribose moieties have been replaced by the high‐affinity RNA analogue Locked Nucleic Acid^™^ (LNA^™^, Exiqon). AntagomiRs bind to mature and precursor miRNAs and thus effectively remove them from the biologically available pool (Elmen *et al*. 2007, Gebert *et al*. 2014). MiRNA sponges constitute an alternative inhibition strategy. Acting after a similar principle, miRNA sponges contain multiple miRNA‐binding sites in their sequence and thus compete for miRNAs (Zhang *et al*. 2013). Modified oligo‐ribonucleotides may be used for miRNA overexpression and in parallel with inhibitors these are convenient tools for modifying cellular miRNA levels in *in vitro* models (Stenvang & Kauppinen 2008, Chen *et al*. 2015).

The Miravirsen antagomiR targeting the liver‐specific miR‐122 (Roche Innovation Center Copenhagen, previously Santaris Pharma) shows feasibility of miRNA inhibition and is a promising translation of basic microRNA research into therapeutic context (Janssen *et al*. 2013, Ottosen *et al*. 2015). MiR‐122 is required for hepatitis C virus (HCV) replication (Jopling *et al*. 2005, 2008, Henke *et al*. 2008), and Miravirsen administration is able to suppress miR‐122 expression and thus prevent HCV replication (Krützfeldt *et al*. 2005, Ottosen *et al*. 2015).

Within metabolism, only few miRNA inhibitors are in development, one of which the N‐acetylgalactosamine (GalNAc)‐conjugated anti‐miR‐103/107 RG‐125(AZD4076), being developed by Regulus Therapeutics and AstraZeneca for the treatment of non‐alcoholic steatohepatitis (NASH) in patients with type 2 diabetes/pre‐diabetes (RegulusTherapeutics 2014, 2015). The current treatment of NASH with thiazolidinediones is commonly accompanied with undesirable weight gain (Musso *et al*. 2012), and there is an unmet need for improved therapy for this disorder. Treatment with RG‐125(AZD4076) is based on its ability to inhibit the activity of miR‐103/107, whose hepatic upregulation causes insulin resistance (Trajkovski *et al*. 2011). AntagomiR‐based silencing of miR‐103/107 in mice was followed by decreased liver triglyceride content and improved insulin sensitivity. RG‐125 (AZD4076) has therefore the potential to acts as an efficacious insulin sensitizer (RegulusTherapeutics 2015).

RG‐125 (AZD4076) is modified by addition of an N‐acetylgalactosamine (RegulusTherapeutics 2015), which targets the oligonucleotide preferentially to hepatocytes via the binding to the liver enriched ASGR1 (asialoglycoprotein receptor 1). This conjugation enhances potency and aids in avoiding cross‐reactivity with similar miRNA families like miR‐15/16, circumventing two of the challenges facing miRNA‐based pharmaceutical agents; delivery and specificity for intended miRNAs. Currently, AstraZeneca initiated dosing in a first‐in‐human Phase I clinical study of RG‐125(AZD4076) at the end of 2015.

MiRNA‐based therapies offer some distinct advantages over other nucleic acid directed therapies: MiRNAs are efficient silencers and in contrast to plasmid DNA or synthetic oligonucleotides, miRNAs occur naturally in the blood stream. As they target multiple mRNAs, resulting synergistic effects could be positive for therapy (Chen *et al*. 2015) and increase the barriers for formation of resistance (Janssen *et al*. 2013, Wang *et al*. 2015a). A low toxicity and good tolerance in antagomiR‐treated patients support the beneficial role of miRNAs in therapy (Janssen *et al*. 2013, Van Der Ree *et al*. 2014).

However, there are multiple important challenges facing miRNA‐mediated treatments: unmodified miRNAs are rapidly degraded (Chen *et al*. 2015), emphasizing the requirement for chemically modified derivatives or encapsulation. Moreover, activation of the innate immunity or neurotoxicity is potential and important side effects, and miRNA inhibitors are restricted in their actions by cellular uptake and incorporation into RISC, and improper dosing can lead to inhibition of unintended targets causing side effects (Lindow *et al*. 2012). Currently, two of the major challenges with miRNA‐based drugs are delivery (Wang *et al*. 2015a) and low tissue specificity (Kwekkeboom *et al*. 2016). Administration of miRNA‐based drug candidates is mostly carried out by injection either intravenously or locally (Obad *et al*. 2011, Shu *et al*. 2015, Wang *et al*. 2015a), but alternative approaches include oral administration, enema formulation followed by gut delivery, topical application and intra‐ocular delivery (Stenvang & Kauppinen 2008, Kwekkeboom *et al*. 2016). Nevertheless, improved strategies for precise and efficient tissue‐delivery are needed.

Thus, miRNA‐based therapy approaches have potential as new and innovative tools in various diseases, but there is currently only few in development for metabolic disease most likely due to the promiscuous nature of the miRNA targets as well as the difficulties of obtaining tissue specificity.

## Biomarkers

MiRNAs are found in bio fluids, such as blood, urine, plasma and saliva (Javidi *et al*. 2014, Arrese *et al*. 2015). Although pure RNA is prone to rapid degradation, miRNAs from bio fluids show a remarkable stability (Mitchell *et al*. 2008). The common explanation is that these miRNAs are contained in exosomes, which are small membrane vesicles of 40–100 nm size (Chevillet *et al*. 2014), which contain DNA, mRNA and proteins in addition to miRNAs (Ban *et al*. 2015). Surrounded by such membrane structures, miRNAs are protected from RNases. The process of miRNA‐secretion in exosomes is still largely unknown. It is unclear, which tissues contribute to miRNA secretion and whether it is the result of active sorting. Furthermore, it is an open question, whether secreted miRNAs have a biological function. But given the low abundancy of miRNAs in exosomes, which might be as low as one copy per exosome (Chevillet *et al*. 2014), a communication function seems unlikely. Moreover, miRNAs in bio fluids may also be stabilized via binding to Ago2 or lipoproteins (Arroyo *et al*. 2011).

Despite these unclear issues, it is well described that the miRNA patterns of bio fluids change under pathological conditions (Mitchell *et al*. 2008, Ortega *et al*. 2013, Yang *et al*. 2014). MiRNA signatures show promise for use in diagnostic or prognostic tests in a variety of diseases, for example cancer (Javidi *et al*. 2014), polycystic ovary syndrome (Sørensen *et al*. 2014) and liver diseases (Arrese *et al*. 2015) amongst others. Advantages of using miRNAs as biomarkers include sensitivity of detection and possibility of multiplexing analyses for increased specificity. A major challenge for the analysis of miRNAs as biomarkers from bio fluids is to establish consequent and robust protocols for pre‐analytical sample handling, miRNA extraction and measurement, which are all important for reliable results (Blondal *et al*. 2013). All taken together, miRNAs can be used as diagnostic tools, but studies for the future should include investigations of large, population based cohorts to establish base‐line values and degree of between subject variation to enable the use of miRNAs for minimal to non‐invasive biomarkers.

## Quantification of miRNA

Even though RNA molecules have been studied for decades, the adaption of standard laboratory techniques to miRNA research is challenging. While both column‐ and chemical‐based RNA isolation techniques are successfully used for mRNA isolation, these have limitations with regard to miRNA (Trevorlstokes, 2012, McAlexander *et al*. 2013, Moldovan *et al*. 2014). For analysis of miRNA levels, two commonly used approaches are platforms based on sequencing and quantitative PCR (qPCR). During the miRNA quality control study (MiRQC), commercial platforms from 12 different providers were assessed and compared by different quality metrics (Mestdagh *et al*. 2014). It was investigated how well platforms perform when faced with challenges such as discrimination between highly similar miRNA sequences or low abundant miRNAs. As it turned out, each platform has specific strengths and weaknesses. The choice of an optimal approach is therefore highly dependent on the experimental setup and goal.

Integration of mRNA and miRNA expression data based on micro‐array analysis or next‐generation sequencing is a convenient method to investigate possible regulatory mechanisms of miRNAs. However, it is important to realize that miRNA targets preferentially repressed at the translational level will not be detected using this approach. Thus, using mRNA arrays to characterize miRNA‐target regulation is likely to miss true targets regulated by translational inhibition, and therefore, this approach is more likely to report false negative findings. Using Ago2 immunoprecipitation to enrich for mRNAs incorporated into RISC, and thus targeted by a miRNA, is an alternative approach to identify miRNA targets enabling identification of targets regulated at the translational level as well.

Analysis, quantification and transparency of miRNA data have become critical steps. A particular drawback is the absence of a standard reference for normalization. The use of snoRNAs, such as U6, is undesirable, because of different stability and biogenesis compared with miRNAs (Vandesompele 2013, Hellemans & Vandesompele 2014). Other strategies, including global mean normalization (Zhao *et al*. 2010) or several stable reference genes (Bustin *et al*. 2009, Mestdagh *et al*. 2009), should be used. To increase analysis transparency, detailed information regarding miRNA sequence and name should be explicitly noted in resulting publications (Van Peer *et al*. 2014), for example using the miR‐tracker software (Van Peer *et al*. 2014).

Identifying target mRNAs of a given miRNA is crucial in understanding the biological context of miRNAs, but nevertheless remains a complex issue. The imperfect base pairing of miRNA‐mRNA duplexes is challenging for software algorithms (Witkos *et al*. 2011). Consequently, most algorithms struggle with a high number of false‐positive results and low accuracy (Ekimler *et al*. 2014). Current prediction algorithms are available as online tools (Fig. 2b) or source code. The starting point is usually the ‘seed region’ from nucleotide 2–7 of miRNAs (Mazière & Enright 2007, Vlachos & Hatzigeorgiou 2013, Peterson *et al*. 2014), which used by RISC to bind mRNAs by Watson‐Crick base pairing to the miRNA (Saito & Sætrom 2010, Peterson *et al*. 2014). Depending on the algorithm, other features are also taken into account: the location of the binding site on the mRNA, sequence conservation across species, free energy of the formed duplex, accessibility of the mRNA binding site, surrounding miRNA‐recognition sites and miRNA expression profiles in the investigated tissue (Saito & Sætrom 2010, Vlachos & Hatzigeorgiou 2013, Ekimler *et al*. 2014, Peterson *et al*. 2014). Different balances of these features can cause discrepancies in the results. Preferably, several algorithms can be combined in the analysis (Witkos *et al*. 2011) followed by experimental validation. Even though bioinformatic target prediction methods have their limits, they can supplement experimental approaches, giving rise to a more efficient identification of miRNA targets. In conclusion, both the analysis and target prediction of miRNAs provides new challenges for wet and dry laboratory.

## Conclusions and perspectives

A large number of miRNAs have been implicated in different facets of the metabolic syndrome and diabetes mellitus, and currently, there are no well‐established or unifying sets of miRNAs characterizing the various subphenotypes of metabolic disease. However, a number of miRNAs appear to affect the function or differentiated state of the pancreatic *β*‐cell, whereas miRNAs in skeletal muscle, liver and adipose tissue constitute different and almost non‐overlapping sets of miRNAs (Table 1). The field of miRNA research is developing rapidly with new tools and models arising. This will enable the further development of miRNA‐based therapeutics for the treatment of metabolic diseases for which there is an unmet need worldwide. Moreover, seeing the impact of regulation of miRNA processing in adipose tissue, it could be highly useful to identify endogenous as well as small molecule regulators of miRNA processing with the potential use of modifying adipose tissue metabolism for treatment of metabolic disease.

A general conclusion is that with many contrasting studies regarding expression regulation and function of miRNAs in different cell types suggests that there is a genuine need for more studies. As many miRNAs appear to have roles in stress response modulation, whereas they are dispensable in the unperturbed state, it will be necessary to investigate the proper models of metabolic stress in order to elucidate their functions. Moreover, because miRNAs often exist in families, an increasingly important approach will be to modulate levels of entire miRNA families or coregulated miRNAs together as well as separately in order to establish their roles in the intact tissue or organ.

This work, and the Symposium on MicroRNAs in Metabolism, from which this review originates, was sponsored by the Danish Diabetes Academy supported by the Novo Nordisk Foundation. We are very grateful for the support from the Invited Speakers of this Symposium for sharing their view on MicroRNA research in Metabolism and for their constructive comments to this review. A full list of Speakers at the Symposium on MicroRNAs in Metabolism can be found in Appendix S1. The authors would like to thank Lena Eliasson, Romano Regazzi, Marcelo Mori, Brendan Egan, Peter Mouritzen, Bader Zarrouki and Hongbin Zhang for critical revision and their constructive comments on this manuscript. Funding: This work is supported by the Danish Diabetes Academy supported by the Novo Nordisk Foundation, Roskilde University and the FSS, DFF¦Danish Medical Research Council 1331‐00033 to L.T.D. The funders had no role in decision to publish or preparation of the manuscript.

## Conflicts of interest

JG, SM and LTD have nothing to declare. SV is currently employed by Novo Nordisk A/S, a pharmaceutical company selling products for treatment of diabetes mellitus.

## Supporting information


**Appendix S1** Speakers at the microRNAs in metabolism symposium, who all contributed to the manuscript.
