# Supplementary material for: MicroRNAs in metabolism
Source: Acta Physiol (Oxf). 2016 Apr 5;219(2):346–61. doi: 10.1111/apha.12681 (PMC5297868; doi:10.1111/apha.12681)
Supplement: Supplementary file 1 — Appendix S1 Speakers at the microRNAs in metabolism symposium, who all contributed to the manuscript. [file APHA-219-346-s001.docx]

**Speakers at the MicroRNAs in Metabolism Symposium, who all contributed to the manuscript:**

Markus Stoffel^1^, Carlos Fernandez-Hernando^2^, Hongbin Zhang^3^, Lena Eliasson^4^, Romano Regazzi^5^, Jonathan Esguerra^4^, Brendan Egan^6^, Paula da Costa-Martins^7^, Marcelo Mori^8^, Agne Kulyte^9^, Jan-Wilhelm Kornfeld^10^, Bruna Brandao^8^, Jo Vandesompele^11^, Bader Zarrouki^12^, Peter Mouritzen^13^

^1^ Institute of Molecular Health Sciences, Swiss Federal Institute of Technology, Zurich, Switzerland, ^2^ Integrative Cell Signaling and Neurobiology of Metabolism Program, Section of Comparative Medicine, Yale University School of Medicine, New Haven, USA, ^3^Section on Integrative Physiology and Metabolism, Joslin Diabetes Center, Harvard Medical School, Boston, MA, USA and Department of Biomedical Sciences, University of Copenhagen, Copenhagen, Denmark, ^4^Department of Clinical Sciences Malmo, Lund University Diabetes Centre, Lund University, Malmo, Sweden, ^5^Department of Fundamental Neurosciences, University of Lausanne, Lausanne 1005, Switzerland, ^6^Institute for Sport & Health, School of Public Health, Physiotherapy, and Sports Science, University College Dublin, Ireland, ^7^Department of Cardiology, CARIM School for Cardiovascular Diseases, Maastricht University, 6229 ER Maastricht, The Netherlands, ^8^Department of Biophysics, Federal University of Sao Paulo, Sao Paulo, Brazil, ^9^Lipid laboratory, Department of Medicine Huddinge, Karolinska Institutet, SE-141 86 Stockholm, Sweden, ^10^ Cologne Excellence Cluster on Cellular Stress Responses in Aging Associated Diseases, Köln, Germany; Max-Planck-Institute for Metabolism Research, Köln, Germany, ^11^Center for Medical Genetics Ghent, Ghent University, Ghent, Belgium, ^12^CVMD iMED, Astrazeneca, Sweden, ^13^Peter Mouritzen, Exiqon A/S, Vedbaek, Denmark
